# The Impact of Mastitis on the Biochemical Parameters, Oxidative and Nitrosative Stress Markers in Goat’s Milk: A Review

**DOI:** 10.3390/pathogens9110882

**Published:** 2020-10-24

**Authors:** Cristiana S. Novac, Sanda Andrei

**Affiliations:** Department of Biochemistry, Faculty of Veterinary Medicine, University of Agricultural Sciences and Veterinary Medicine, Cluj-Napoca 400372, Romania; sandrei@usamvcluj.ro

**Keywords:** goat, mastitis, biochemical parameters, oxidative stress

## Abstract

Goat mastitis has become one of the most frequently diagnosed conditions in goat farms, with significant economic impact on the dairy industry. Inflammation of the mammary gland poses serious consequences on milk composition, with changes regarding biochemical parameters and oxidative stress markers. The aim of this paper is to present the most recent knowledge on the main biochemical changes that occur in the mastitic milk, as well as the overall effect of the oxidative and nitrosative stress on milk components, focusing on both enzymatic and nonenzymatic antioxidant markers. Mastitis in goats is responsible for a decrease in milk production, change in protein content with pronounced casein hydrolysis, and reduction in lactose concentration and milk fat. Milk enzymatic activity also undergoes changes, regarding indigenous enzymes and those involved in milk synthesis. Furthermore, during mastitis, both the electrical conductivity and the milk somatic cell count are increased. Intramammary infections are associated with a reduced milk antioxidant capacity and changes in catalase, lactoperoxidase, glutathione peroxidase or superoxide dismutase activity, as well as reduced antioxidant vitamin content. Mastitis is also correlated with an increase in the concentration of nitric oxide, nitrite, nitrate and other oxidation compounds, leading to the occurrence of nitrosative stress.

## 1. Introduction

Goat milk and dairy products, such as yoghurt and cheese, have recently become increasingly popular among consumers due to their composition and nutritional value, which is different from cow milk, leading to positive effects on human health. Goat milk differs from cow milk mainly in that it consist of higher oligosaccharide content, a higher level of medium chain triglycerides, and higher β-casein and lower αs1-casein content when compared to cow milk [1].

Mastitis is defined as inflammation of the mammary gland, which most often occurs as a result of infections with different pathogenic agents, such as bacteria, fungi or viruses, and it is also associated with poor hygiene conditions in dairy farms [2]. Infectious processes that take place in the goat mammary gland are accompanied by numerous changes in milk composition. These changes target both the main biochemical parameters and the oxidative stress markers. Unlike other species, goat milk composition can also be affected by non-infectious factors, such as breed, age, stage of lactation, estrous cycle or feeding [3]. Inflammation of the mammary gland produces a series of changes that vary depending on the severity of lesions, the degree of damage of the udder parenchyma, and also the pathogen causing the infection. Thus, the main changes are as follows: a decrease in milk production, both in the case of subclinical and clinical mastitis; lower lactose content; an increase in the concentration of whey proteins; a change in the proportion of caseins; and decrease in lipid concentration [3]. Moreover, the enzymatic activity in the mammary gland undergoes significant changes. Indigenous enzymes increase during inflammatory processes, while enzymes involved in milk synthesis are characterized by a lower activity [4]. Milk enzymes are divided into two broad categories, non-lysosomal and lysosomal. The main non-lysosomal enzyme is lactate dehydrogenase (LDH), whose activity increases in mastitis, while the second category of enzymes includes β-galactosidase, N-acetyl-β-glucosaminidase (NAG), α-mannosidase and β-glucuronidase, which also increase during inflammatory processes [5]. Certain enzymes of blood origin, such as plasmin, are characterized by a very high activity in mastitis. Another indicator of mastitis is the milk somatic cell count (MSCC), which increases in the case of inflammation [6].

Furthermore, intramammary infections lead to the accumulation of reactive oxygen species, with the occurrence of oxidative stress in the udder tissue. To counteract these effects, the milk possesses its own antioxidant systems, which play an important role in maintaining its quality. The milk antioxidant activity is due to the presence of enzymes, such as catalase, lactoperoxidase, glutathione-peroxidase, xanthine oxidase or non-enzymatic compounds, vitamins and provitamins. Intramammary infections have consequences on the antioxidant activity, thus, some compounds can be used as indicators of mastitis, such as catalase, lactoperoxidase or glutathione-peroxidase [7,8].

The aim of this paper is to present the most recent knowledge about goat mastitis and how intramammary infections in this species affect both the milk biochemical composition and the oxidative stress markers. Moreover, this review will briefly present the correlation between different pathogenic agents involved in the etiology of mastitis and the changes that occur in the milk.

## 2. Changes in Milk Biochemical Parameters

As mentioned above, goat milk composition can be affected by a wide range of factors, which can be divided into noninfectious and infectious factors. The first category includes the following: breed, age, stage of lactation, estrous cycle and feeding. On the other hand, infectious factors include inflammatory reactions in the mammary gland caused by various pathogens, such as bacteria, fungi or viruses [9,10]. The onset of inflammation has negative consequences on the quantity, quality and milk processing properties. Mastitis is the most frequently diagnosed disease in milk-producing species, both small and large ruminants, being the main cause of economic losses in the dairy industry worldwide. Beyond animal health and food safety matters, mastitis is also a problem for the dairy industry due to changes in milk composition [11]. Staphylococci, streptococci and *Escherichia coli* are the most common bacterial agents involved in the etiology of mastitis in cattle, sheep and goats [9,10,12]. In small ruminants, subclinical mastitis has a higher prevalence than clinical mastitis, with a percentage ranging between 5% and 30% [13] and may even go up to over 50% [14], while the prevalence of clinical mastitis is lower than 5% [13,15]. In general, subclinical mastitis in goats is most often caused by non-aureus staphylococci (NAS), whilst in the etiology of clinical mastitis, the following pathogens are involved: *Staphylococcus aureus*, NAS, members of the *Enterobacteriaceae* family, *Corynebacterium* spp., *Pseudomonas* spp. and, *Mycoplasma* spp., but also fungi, such as *Aspergillus fumigatus* [12,13,16,17].

Mastitis is responsible for producing certain changes in the mammary tissue that depend on the severity of lesions and the degree of damage of the udder parenchyma, but an extremely important factor is represented by the pathogen causing the infection [3]. The changes induced by mastitis in the milk biochemical parameters have been thoroughly studied for cows, while the scientific data regarding goat or sheep milk are scarce compared to the former. However, in all milk-producing livestock, mastitis decreases milk production and is responsible for unfavorable changes in milk composition, such as the reduction in fat, protein, casein and calcium content, with a simultaneous increase in the concentration of whey proteins, sodium and chlorine [18]. Moreover, the activity of certain enzymes in milk such as lipases, proteases or plasminogen increases, adversely affecting its technological properties. It should be noted that the decrease or increase in these parameters may be more or less obvious depending on the species [18]. Most often the inflammation of the mammary gland is accompanied by the multiplication of bacteria in milk and the increase in MSCC. Both indicators play a key role in assessing milk quality [18,19].

### 2.1. Milk Production

Both clinical and subclinical forms of mastitis either have negative effects on milk production with major economic consequences. Although the decrease in milk yield is often associated with the destruction of alveolar epithelial cells, this reduction in milk secretion can also occur as a result of so-called blockages of the mammary ducts or clogged ducts, as acute infections lead to the accumulation of bacteria, neutrophils and also fibrin in the ducts. Following the clogging of the ducts, bacteria multiply and necrosis foci appear. As the infection progresses, neutrophils invade the ductal system, the epithelium becomes hypertrophied, and the connective tissue is very pronounced, narrowing the lumina and thus blocking the milk flow. Similarly, the systemic consequences of mastitis that interfere with feed consumption, digestion processes or blood flow to the mammary gland significantly reduce milk production [20].

Regarding subclinical mastitis in goats, quantifying milk loss is a complicated task, regardless of the pathogen involved. Studies conducted on goats which were focused on the effect of intramammary infections on dairy production in intensive growth systems have presented rather controversial results. Thus, mastitis was associated with lower values [3], similar [21,22] or even with an increase in milk production in sick goats compared to healthy ones [23].

Moreover, the variation in milk production also depends on the pathogenic agent involved in the intramammary infection. Thus, studies show that coagulase-positive staphylococci (CPS) are associated with a significant decrease in milk secretion, whereas this observation is not available for NAS [17,24]. On the other hand, a study conducted by [3] demonstrated that certain NAS species, such as *Staphylococcus epidermidis*, *S. simulans*, *S. caprae*, *S. chromogenes* or *S. xylosus*, can induce changes in milk production with a decrease in milk secretion. Moreover, the same observations were made in the case of mastitis caused by NAS in sheep, the decrease being more significant in this species, reaching up to 30%, compared to goats where the reduction of milk production is not so significant. In sheep, for example, the so-called “milk drop syndrome” is mainly caused by mammary infections, especially subclinical ones [25,26,27]. In goats, infections caused by Gram-negative bacteria, as well as *Mycoplasma* spp., have major consequences on milk yield, drastically reducing production [17,24,28].

### 2.2. Milk Proteins

Similar to cow milk, the composition of goat milk changes during infectious inflammatory processes. One of biochemical parameters that suffers variations is represented by proteins. Thus, total proteins may vary to a small extent, but the changes are minor. One study showed that there are no statistically significant differences between the value of total proteins in goat mastitic milk compared to the healthy milk [3]. More recent studies show that mastitis produced by major pathogens such as *Mycoplasma* spp. does not have a substantial effect on milk quality, including proteins [28]. Furthermore, whey proteins and albumin concentration increase significantly in the infected mammary gland, as well as the concentration of IgG. Increased values are also observed in the case of lactoferrin, a compound with bacteriostatic activity, which could explain its high concentration [3,10].

In a study focused on experimentally induced endotoxin mastitis with lipopolysaccharides in goats, no significant changes were observed in protein expression patterns. Therefore, caseins were not profoundly affected, and only minimal changes in serum albumin concentration were observed, which is known to be secreted in cow’s milk with colibacillary mastitis. These results were obtained at an interval of 18, 24 and 48 h post-inoculation. According to the authors, this observation may indicate that the damage to the blood–milk barrier caused by endotoxins released during colibacillary mastitis in goats is not as significant compared to large ruminants [29].

Another important group of proteins that may undergo changes or variations during these infectious inflammatory processes is caseins. In goat milk, the amount of casein varies between 16 and 26 g/L, the main casein being represented by β-casein [30]. Bacterial infections are known to affect the structure of casein micelles. These micelles are synthesized in different proportions in the epithelial cells of the mammary gland and can undergo proteolysis processes following the activity of either indigenous or bacterial enzymes [31,32]. Plasmin is one of the major enzymes found in milk, and its activity has been extensively studied over time. This enzyme belongs to the proteases class and originates in the blood, its inactive precursor being plasminogen. The latter is then activated locally, turning into plasmin, responsible for the degradation of fibrin, casein and various components of the extracellular matrix. During mastitis, the activity of blood enzymes increases, thus explaining the increased activity of plasmin in the udder [4,10]. Regardless of the bacterial species involved, the activity of plasmin in milk from an infected mammary gland is significantly increased (two-fold) compared to a healthy mammary gland, as studies conducted on bovine milk show [33]. The plasminogen activator/plasmin system found in milk is identical to the one in the blood in terms of amino acid sequence, but the same observation is available for both biochemical and immunological properties [34]. Plasmin is responsible for the hydrolysis of casein, especially β-casein, by cleaving it into different subtypes of γ-caseins and protease-peptones, which in turn induce a decrease in the lactation curve. It has been shown that plasmin activity increases during intramammary infections, leading to increased protease-peptone levels [26,31,32,33,35]. In sheep, for example, mastitis leads to an increase in the casein hydrolysis products, thus along with the increase in the protease-peptones concentration in milk, the percentage of β-casein decreases. On the other hand, the proportion of α-caseins and κ-caseins in sheep milk does not appear to be significantly affected by these endogenous proteolysis processes [36]. Such proteolytic processes have also been reported in goat milk, where β-casein is more susceptible to hydrolysis, similar to sheep milk. In addition to this category of caseins, another class of easily hydrolyzable proteins is αS2-caseins. Proteolysis of these compounds is associated with an elevated MSCC and increased number of neutrophils present in the mammary gland. Neutrophils represent the main source of proteolytic enzymes such as plasmin, elastase or cathepsin B, which hydrolyze caseins [29].

Goats diagnosed with subclinical mastitis or in the final stage of lactation have an increased level of plasmin in milk, which is also associated with an increased MSCC and a high concentration of gelatinase [37]. The high degree of plasmin activation reduces milk production, leading to changes in the secretion of organic compounds, thus affecting milk composition, probably due to structural changes in the casein molecule that affect its ability to agglutinate. A moderate increase of 30–50% in plasmin activity leads to a decrease in milk production, but with a higher increase in the protein and lipid content at the same time. The hydrolysis of caseins takes place under these conditions, with β-casein breaking down into peptides, leading to a reduction of milk secretion in cattle and goats. On the other hand, there are cases in which the level of protein and fat is lower in the infected gland compared to the healthy udder tissue. Such response was observed when plasmin activity was significantly increased (twice or more), as is the case with milk stasis [3].

Proteolysis can also occur due to the activity of bacterial enzymes [31,32,38]. Thus, studies show that *E. coli* would be able to degrade caseins in vitro and that the hydrolysis of these proteins is more pronounced in experimental *E. coli* infection compared to mastitis caused solely by lipopolysaccharides inoculation. The in vitro activity of the proteases released by this bacteria is, however, quite low. To explain casein lysis, it has been hypothesized that these proteolytic enzymes are secreted in a much higher concentration in vivo or that *E. coli* could have an indirect effect on casein hydrolysis by increasing the secretion of endogenous proteases or factors that produce the maturation of somatic cells [10,38]. Studies on experimental infections with *E. coli*, *Streptococcus uberis* and *Streptococcus agalactiae* have shown that these pathogens are responsible for inducing apoptosis and cell proliferation. *E. coli* and *Staphylococcus aureus* both cause necrosis of the mammary epithelium, especially in cases of severe mastitis [39]. Furthermore, *E. coli* secretes proteolytic enzymes that contribute to the degradation of the extracellular matrix. Thus, it has been shown that certain enzymes, such as gelatinases (especially matrix metalloproteinases, MMP-2 and MMP-9) that degrade the basal membrane and interstitial proteins, have 300 times higher values in cow’s milk with colibacillary mastitis compared to milk from healthy animals [10].

Other pathogens that secrete proteases which are able to degrade caseins are CPS, such as *Staphylococcus aureus*, as well as NAS. In bovine, the quarters affected by *S. aureus* have a smaller alveolar area and a larger stromal area compared to healthy quarters. Thus, the infected section has a reduced synthesis capacity. This observation could apply to other species of milk-producing ruminants [10].

### 2.3. Carbohydrates

Lactose is the major carbohydrate in goat milk, made up of a glucose and a galactose molecule [30]. Goat milk is characterized by an average lactose content of 4.16 g/100 mL, which is lower than cow milk (4.76 g/100 mL). In addition to this disaccharide, goat milk also contains oligosaccharides, glycopeptides and glycoproteins, the oligosaccharides playing an important role as prebiotic compounds that stimulate the multiplication of probiotic bacteria in the intestinal flora [40,41].

The evolution of subclinical mastitis in the goats is characterized by a significant decrease in lactose compared to milk from a healthy mammary gland [3]. This reduction in lactose concentration can also be seen in sheep or cows, as a result of increased plasmin activity. As previously mentioned, the degree of plasmin activation not only causes a reduction in milk secretion, but also produces changes in the secretion of certain organic compounds, such as lactose [3,42].

### 2.4. Lipids

Lipids represent the most important constituent in milk, being responsible for the specific flavor and taste of goat milk and other dairy products such as yoghurt and cheese. These compounds are present as globules in milk, which are smaller compared to cow fat globules [30]. This is the reason why goat milk has a higher digestibility and it is recommended to patients with gastro-intestinal disorders or allergies to cow milk [43].

Lipids are well represented in goat milk, but their percentage varies depending on breed, stage of lactation, season and feeding. On average, goat milk contains 4.5% lipids, and what is characteristic of this species is the higher content of short- and medium-chain fatty acids than cow milk [41,44].

Goat intramammary infections have consequences on milk fat percentage, but literature studies present controversial results; one research showed that the percentage of lipids decreases in the case of mastitis in goats, unlike sheep, where no significant differences are observed between the fat content of the affected udder compared to the healthy one. However, in interpreting these parameters, it is recommended to take into account the stage and number of lactations, because as we approach the end of lactation, the fat and protein content increases [25,45]. On the other hand, other data show that the percentage of lipids in milk from a mammary gland affected by mastitis does not differ significantly from healthy milk [3,22,46].

### 2.5. Enzymes

In regard to indigenous enzymes, milk is considered to be an extremely complex solution. Thus, more than 60 enzymes have been identified in the milk of different mammalian species, enzymes that play an important role in regulating lactogenesis. Besides this, some enzymes are essential components of the milk antioxidant and immune defense system [8,47]. Milk, however, is not a homogeneous solution of enzymes, due to the fact that they are specifically associated with one or more of its five phases: whey, fat globules, casein micelles, membrane vesicles and milk cells (lymphocytes, polymorphonuclear cells and macrophages). According to some authors, the distribution of enzymes in milk is closely related to the way they were secreted. The mechanisms through which these compounds are released into milk are not yet fully described [47].

The changes induced by the evolution of mastitis depend on the pathogenicity of bacteria, but also on the degree of damage to the udder tissue, especially the epithelial region. Thus, the main changes in the mammary gland include the following: transport of ions, proteins and blood enzymes into the milk due to increased permeability; invasion of phagocytic cells in milk secretion; and last but not least, a reduction in the synthesis capacity of the gland. Therefore, the concentration of certain enzymes changes once the pathological process is installed. The activity of some indigenous enzymes increases during inflammation, while the enzymes involved in the process of milk synthesis have a lower activity [4].

The synthesis of polymorphonuclear cells (PMN) and macrophages is one of the organism’s essential mechanisms of defense against clinical and subclinical mastitis. For this reason, the rise in MSCC is also observed, these cells being represented in a very large proportion by PMN. During inflammatory processes, PMN secrete hydrolytic enzymes and other compounds. The enzymes may be non-lysosomal, such as lactate dehydrogenase, or lysosomal enzymes, such as β-galactosidase, N-acetyl-β-glucosaminidase, α-mannosidase and β-glucuronidase. Among the lysosomal enzymes, N-acetyl-β-glucosaminidase and β-glucuronidase are released more intensely during intramammary infections [48]. Moreover, the activity of certain enzymes of blood origin is higher, as is the case of plasminogen activated to plasmin, an aspect discussed above. The main milk enzymes and their variations during mastitis will be presented in the following paragraphs.

#### 2.5.1. Lactate Dehydrogenase

Lactate dehydrogenase (LDH) is a ubiquitous enzyme which is present in the cytoplasm of all cells and tissues in the animals, but it is also found in bacteria and yeasts. This enzyme is part of the glycolytic pathway, and its role is to mediate the oxidative/reductive connection between pyruvate and lactic acid [49]. LDH is a non-lysosomal enzyme whose activity changes during mastitis, with a significant increase in milk, as multiple studies on bovine milk show [50,51,52]. These changes are correlated with the destruction of udder tissue caused by intramammary infections. Infectious processes increase the permeability of microcapillaries through chemical mediators, such as histamine, prostaglandin, quinines and oxygen free radicals in inflammatory cells. Higher levels of LDH in mammalian milk compared to blood levels show that blood serum is not the only source of this enzyme [53,54]. LDH can be released during the immune response by the udder’s parenchyma cells and leucocytes [55]. These aspects were observed in both sheep and goat milk. Studies have shown that with increasing LDH activity, there is a rise in alkaline phosphatase and aspartate aminotransferase levels in milk [53,56]. LDH could be considered a reliable indicator for the intensity of the inflammatory process in the mammary gland, especially in the case of subclinical mastitis, but in order to establish a correct diagnosis, the stage of lactation and parity must be taken into account. Thus, in the first week after calving, the enzyme level is very high; with the increasing number of lactations, the activity of this enzyme is higher, an aspect which has been also observed in cattle. Moreover, highly pathogenic bacteria lead to a marked increase in LDH activity compared to bacterial agents with lower pathogenicity or those considered minor pathogens [55,56]. Among LDH, alkaline phosphatase and aspartate aminotransferase, LDH is considered the most reliable indicator for the detection of intramammary infections in small ruminants [56]. The activity of this enzyme can be measured using simple spectrophotometric methods or fluorescence spectroscopy, the latter being a more accurate and suitable method for the analysis of heterogeneous liquids such as milk [49].

#### 2.5.2. N-Acetyl-β-D-Glucosaminidase

N-acetyl-β-D-glucosaminidase (NAGase) belongs to the category of lysosomal enzymes. Once the infectious process is installed, significant changes are noted regarding its concentration in milk, a phenomenon observed in different species of milk-producing ruminants. NAGase originates in phagocytic cells, more precisely in neutrophils, so these lysosomal enzymes are known to be reliable indicators of inflammation. During phagocytosis and cell lysis, NAGase is released into the milk in high amounts. This enzyme can also be released by damaged epithelial cells. The activity of this enzyme is also correlated with MSCC [4]. Furthermore, NAGase catalyzes cellular reactions, a phenomenon which plays an important role in helping the immune system fight against pathogenic agents [55]. Studies have confirmed this observation, showing that the addition of this enzyme leads to a bacterial reduction, therefore, NAGase has a bactericidal effect [57]. Milk from goats with mastitis is characterized by a higher NAGase activity compared to milk from a healthy mammary gland. In the past years, numerous studies have confirmed this hypothesis. The increase in the enzymatic activity is also observed in cases of subclinical mastitis, especially in those caused by NAS [3,46,58,59]. Research aimed at evaluating the impact of mammary gland infections on the enzymatic activity of milk in the first part of lactation in goats (first 6 weeks) demonstrated that the NAGase level is high in early lactation and that pathogens do not alter the enzymatic activity during the studied period. The activity of NAGase can be determined using a fluorescence spectroscopic method [55].

#### 2.5.3. β-Glucuronidase

β-glucuronidase is a lysosomal enzyme that has proven to be, according to some authors, the most relevant marker in the development of the inflammatory process in the mammary gland, especially if it is combined with MSCC and the result of California Mastitis Test (CMT) [48]. This enzyme is part of the hydrolase family, and it is also thought to be involved in the organism’s innate immune response. β-glucuronidase is responsible for splitting glucuronides in glucuronic acid and its aglycone [55]. Unlike NAGase, β-glucuronidase activity is not likely to be influenced by the stage of lactation. In the case of mastitis, the activity of this particular enzyme is positively correlated with the activity of other enzymes, such as LDH, NAGase or alkaline phosphatase, as well with MSCC, infectious processes causing enzyme levels to increase [55]. In goat mastitis, β-glucuronidase is the most selectively released enzyme [48]. However, studies suggest that the use of β-glucuronidase activity alone may not be a reliable method to detect mastitis [60]. Recently, some authors developed an optimized fluorometric method for a more precise determination of β-glucuronidase in ruminant milk to be used along with other indicators for mastitis [60].

#### 2.5.4. Metalloproteinases

Metalloproteinases (MMP) are zinc-dependent endopeptidases with broad substrate specificity, which are synthesized in a latent form as inactive zymogens that must undergo activation through proteolytic processes or they can suffer conformational changes induced by oxidative stress [61]. MMP are a group of more than 20 metallopeptidases which include gelatinases, collagenases, stromelysins, matrilysins and membrane-type MMP [62].

Matrix metalloproteinase (MMP-9) is a zinc-dependent enzyme that plays a particularly important role in extracellular matrix degradation, tissue remodeling and also inflammation It is secreted by various cell types, such as capillary endothelial cells, neutrophils and macrophages [37,62]. The correlation between this enzyme and some goat milk parameters, such as somatic cells, is not fully understood. A recent study demonstrated that MMP-9 activity is predominantly expressed in the spleen, intestine and mammary gland, so that the MSCC in this species is directly proportional to the activity of this enzyme. Moreover, *S. aureus* could significantly influence metalloproteinase levels in mammary epithelial cells, causing an increase in the enzymatic activity. It has been hypothesized that during inflammatory processes, MMP-9 would alter vascular permeability and the blood–milk barrier, stimulating the transport of white blood cells to the inflammation site. The same study also showed that an inhibitor of this enzyme, SB-3CT, stimulates apoptosis and inhibits the proliferation of mammary epithelial cells, thus, MMP-9 could be considered both a parameter capable of reflecting MSCC and a regulatory factor for apoptosis in goat mammary epithelial cells [37]. Furthermore, studies on bovine milk have reported that the elevated MSCC induced a significant increase in the level of plasmin and milk gelatinases, thus suggesting that there is a certain correlation between the activity of MMP-9 and MSCC [63]. The expression of this enzyme is increased in somatic cells in goat milk during intramammary infections due to the fact that the organism recruits immunologically competent cells at the site of inflammation, causing MMP-9 to degrade the extracellular matrix, consequently making this enzyme a possible tool for diagnosing early mastitis [37].

### 2.6. Electrical Conductivity

The inflammation of the mammary gland is also responsible for modifying the electrical conductivity of milk, which has also been observed in goats. This parameter is currently used in cattle in the detection of subclinical mastitis, where it is of course correlated with other indicators. It is an advantageous method because it is not extremely costly, there is the possibility of automation in dairy farms, and it is also a quick and reliable method [64,65]. This parameter is determined by the concentration of cations and anions. As general knowledge, mastitis increases the concentration of Na^+^ and Cl^−^ in the milk, which then causes the electrical conductivity to rise [66]. Similar to the results obtained by studies conducted on cow milk, research on different goat breeds has shown that this indicator increases in the case of mastitis, especially when both halves of the mammary gland are affected. The increase in electrical conductivity is accompanied by significant increase in chlorine content [65,67]. In regard to goat breeds, results are controversial, therefore, in a study carried out on Saanen and Alpine goats, it was observed that the electrical conductivity of milk increased in the case of mastitis in Saanen breed, but surprisingly decreased in Alpine goats [67]. Furthermore, it has been noticed that the sodium:potassium ratio is also elevated during intramammary infections [68]. A marked increase in electrical conductivity is observed especially when the inflammation affects the mammary gland bilaterally, in which case both the chloride content and the sodium:potassium ratio increase substantially. On the other hand, if the infection is unilateral, the increase in conductivity is low, while chloride undergoes a marked rise. This phenomenon suggests that the blood–milk barrier is much more affected in the case of bilateral gland damage compared to unilateral one [65]. Thus, in the case of mastitis, the permeability of blood vessels, especially of the capillaries in the udder, increases, facilitating the transport of electrolytes from the blood directly into the milk [69]. One study, which aimed to evaluate the effect of intramammary infections on the electrical conductivity of goat milk from the Murciano-Granadina breed, demonstrated that the microorganisms that led to significant changes were *Staphylococcus aureus* and Gram-negative bacteria. Other pathogens, such as NAS and *Streptococcus* spp., did not increase milk conductivity [65].

In cows, this parameter seems to be affected by other factors as well besides mastitis, such as lactation or parity, so it is not recommended to make a singular measurement of the milk conductivity in order to establish a diagnosis. Good results in detecting mastitis were obtained when this parameter was measured daily, and variables such as temperature or milk production were taken into account [65]. In goats, similar to the aspects observed in cattle, other factors besides infections have an effect on the electrical conductivity of milk. Among these factors, we list stage of lactation, parity or milk composition. Regarding parity, the electrical conductivity of milk from multiparous goats is higher than that of primiparous animals in the absence of mastitis. Moreover, multiparous goats have an increased milk conductivity once the lactation progresses, because there is a higher transfer of minerals from blood into the milk at the end of the lactation period due to increased permeability. Therefore, the measurement of this parameter is recommended to be conducted periodically, its correlation with other examinations (for example CMT or MSCC) being essential in order to establish a correct diagnosis of mastitis [65,68].

### 2.7. Milk Somatic Cells

MSCC is widely used for assessing milk quality and is considered a useful tool to monitor milk hygiene [42,70]. Currently, the MSCC is a valuable indicator used in the diagnosis of mastitis in cows, especially in the case of subclinical mastitis, which does not evolve with visible signs of mammary gland damage, but only with changes in milk composition [71]. Thus, as required under EU Regulation 853/2004, raw cow milk must not exceed 400,000 cells/mL. This result is based on a geometric average over a 3 month period, with at least one sampling per month. What exceeds this number is considered mastitic milk, unfit for human consumption. In recent years, such a standardization of goat milk has also been attempted, but the physiology of the mammary gland and the mechanisms of lactogenesis in this species make it difficult or impossible to achieve, therefore, no generally accepted threshold has been established [59]. In goat milk, a large number of cytoplasmic particles originating from the apical portion of the secretory cells are found physiologically, due to the fact the secretion of the mammary gland is apocrine. Although the vast majority of these particles are anucleated, some of them may contain nucleus fragments, leading to a false increase in MSCC. This is due to the fact that the cytoplasmic particles found in milk are similar in size to the somatic cells. Therefore, somatic cell counting methods based on DNA detection are mandatory in order to obtain a correct result [71,72]. Some authors proposed flow cytometry methods to make a quick differential caprine cell counts, using DNA-specific fluorescent dyes to avoid somatic cells and cytoplasmic particles from overlapping [72]. Although milk secretion in sheep also has an important apocrine component, the concentration of these particles is lower compared to the goat [3,71]. Their average concentrations in goat milk is 150 × 10^3^/mL, whereas for sheep this is 15 × 10^3^/mL [12]. Nevertheless, in both goats and sheep, the MSCC of an uninfected mammary gland is higher compared to that of a healthy mammary gland in cattle [3,59]. This particular high variability of MSCC in goats is caused by intramammary infections, but nevertheless by physiology [59]. In this species, neutrophils (PMN) comprise the predominant cell type in milk from both infected and healthy mammary glands. In the absence of mastitis, PMN represent 45–74% of the milk somatic cells, whereas mastitic milk contains up to 71–86% PMN. Furthermore, 9 to 20% [71] or 22% [67] of the somatic cells in a healthy mammary gland are represented by lymphocytes, whereas in infected glands, the percentage of lymphocytes is lower, up to 11%. Macrophages constitute 15–41% of the somatic cells in milk from uninfected gland and between 8 and 18% in affected gland [71]. Due to the fact that neutrophils represent the first line of attack against pathogens, goats seem to be more resistant to naturally occurring infections (an infection which occurs in a natural habitat and natural conditions, without being experimentally induced) of the mammary gland compared to sheep and cows [73]. This resistance is due to the fact that neutrophils represent up to 74% of milk somatic cells (compared to only 2–28% in ewes [71] and 5–20% in bovine milk [74]), these cells playing a key role in phagocytosis and destruction of bacteria. Moreover, some authors consider that healthy ruminants with lower MSCC values are more susceptible to mastitis compared to healthy animals with high MSCC, suggesting that PMN play a protective role against intramammary infections [73]. Milk somatic cells also include eosinophils and epithelial cells as well, but they are found in a very low percentage [12,42]. The presence of epithelial cells in the milk is not associated with an ongoing inflammation; they originate from the alveolar epithelium and the mammary gland ducts. Their presence in milk is physiological, as it demonstrates the regeneration of the mammary epithelium [74]. A very interesting aspect is the fact that the chemotactic factors that attract PMN to the healthy mammary gland are different from those which are involved in the infectious processes. Moreover, some studies have shown that not only neutrophils and macrophages play an important role in defense against pathogens, but also that eosinophils would be a key component in the fight against pathogenic bacteria [19]. It is also estimated that during mastitis, about 30% of neutrophils in milk undergo cell death, either by necrosis or apoptosis, compared to only 8% of neutrophils in the blood. Similarly, the amount of gelatinase released by milk neutrophils is lower compared blood. Gelatinase is known to mediate the recruitment of neutrophils into the mammary gland during the inflammatory process [75]. Moreover, during these infections, it seems that the PMN in milk have a lower activity than those in the blood, so they are less functional, leading to a decrease in the phagocytic capacity [73].

Recent studies discuss the applicability of direct and indirect methods of cell counting to diagnose goat mastitis, but a significant correlation between the increase in MSCC and the evolution of a mammary infection has not been established [55,76]. However, similar to cows, mastitis is a major cause of the increase in MSCC in goats and the decrease in milk production, but the correlation between MSCC and bacterial infections is not as simple as in cattle. This is mainly due to the fact that in goats, there are both non-infectious and infectious factors that contribute to the increase in somatic cells. The non-infectious factors include intrinsic and extrinsic factors [74].

#### 2.7.1. Intrinsic Factors

Intrinsic factors depend on the animal and are mainly represented by stage of lactation, parity, prolificacy, breed and milking time. One of the intrinsic factors that affects MSCC in goats is milking time. Thus, in this species, it has been shown that the MSCC is between 17 and 78% higher in the evening than in the morning milk. Some authors explain this phenomenon in terms of the dilution effect, because the amount of milk obtained in the morning is 35 to 69% higher than that obtained in the second half of the day [74,77].

The stage of lactation has consequences not only on MSCC, but also on several parameters and constituents of milk which have been previously discussed. The MSCC has an increasing physiological trend along with the progression in the production period in dairy goats [12,42,71]. Therefore, a study conducted by [78] showed that MSCC went from an average of 200 × 10^3^ cells/mL at 15 days of lactation to 500 × 10^3^ cells/mL at 285 days. This increase in cell count with the stage of lactation seems to be inversely proportional to milk production [74]. In the first week after calving, the MSCC level is particularly high, so it is recommended this observation to be taken into account and the values in the first week postpartum to be considered and interpreted separately [55]. However, for later parities for example, MSCC decreases with advanced lactation [78].

MSCC in goats is significantly influenced by parity, an aspect confirmed by the increase in PMN along with the number of lactations [79]. A progressive increase was observed regarding first lactation MSCC, from 140 × 10^3^ cells/mL to 600 × 10^3^ cells/mL for the fifth lactation, as a study conducted on healthy Murciano-Granadina goats showed [71]. Furthermore, the influence of parity on the MSCC depends on the mammary gland health status, thus on the absence or presence of an intramammary infection. Age is considered to be a parameter that influences MSCC especially in the case of mastitis [74]. The influence of age can be associated with a longer exposure of older animals to various pathogens, but it can also be associated with chronic mastitis installed during previous lactation, which either remained untreated or did not completely heal [74,80]. 

The prolificacy or number of kids is another parameter that influences goat MSCC. Some studies have shown that a high count, up to 1666.9 × 10^3^ cells/mL [74], is recorded in animals with more than one kid, compared to those with simple birth [71,73].

Animal breed is known to influence this milk indicator, but it seems that differences between breeds are rather associated with variations in the mammary gland health and with the level of production [74]. Literature is still scarce in genetic data to prove significant correlations between breed and somatic cells. However, a very recent study conducted on several goat breeds showed that milk from Saanen goats is characterized by a greater MSCC compared to Mediterranean breeds, such as Maltese, Jonica, Rossa Mediterranea, Girgentana, and Garganica breeds. This observation applies to the whole lactation period [81].

The estrous cycle significantly influences MSCC in goats, whether it is natural or induced. Thus, during this period, the MSCC is higher. This increase is accompanied by a slight decrease in milk production [74]. However, this phenomenon is temporary, because in the luteal phase the somatic cells begin to decrease in number, suggesting that this initial increase may be caused by estrogen. In estrous, the udder presents an increased sensitivity to this hormone, which induces desquamation of the epithelial cells [82]. In an experimental study conducted by [82] on Saanen goats, MSCC revealed higher values at estrous, 7195 ± 672 × 10^3^ cells/mL and lower values toward the luteal phase, 1694 ± 672 ± 10^3^ cells/mL. Some authors consider that the effect of estrous would depend on the health of the mammary gland, suggesting that heat period may cause a higher increase in MSCC in the infected gland compared to an uninfected one [12].

#### 2.7.2. Extrinsic Factors

The type of milking can influence MSCC in goats, although literature studies present contradictory results. Some authors claim that the somatic cell count is lower in milk obtained by mechanical milking, while others obtained either data suggesting the opposite or did not find significant differences between mechanical and manual milking [74]. A recent study conducted on Saanen goats showed that the type of milking influenced both the physical parameters of the mammary gland and the milk characteristics. Therefore, mechanical milking was associated with a higher MSCC count (0.787 × 10^6^ cells/mL) compared to manual milking (0.350 × 10^6^ cells/mL) and, nevertheless, a greater consistency of the mammary gland was reported in mechanical milked goats [83]. Furthermore, some authors established a link between the use of automatic valve cups during mechanical milking and the risk of mastitis, with a higher MSCC at the beginning of lactation [84]. Additionally, another study proved that mechanical milking is associated with higher risk of bacterial positivity compared to manual milking and a 1.53 times higher risk for *Streptococcus uberis* infection. On the other hand, manual milking has been correlated with a high risk of *Staphylococcus caprae* infection [80].

Changes in goat MSCC are also due to the effects of feed. An increase in this milk parameter may be due to an unbalanced feed ration, which leads to metabolic disorders such as acidosis or alkalosis. Animals affected by either of these conditions have lower milk production associated with a higher number of somatic cells [85]. On the other hand, supplementing the ration with protein concentrates also leads to an increase in MSCC. Overall, a balanced diet is considered to be the most appropriate to keep this parameter at low values [74]. 

Farming system also influences MSCC, the semi-intensive system being associated with a higher value of this parameter compared to the intensive one, but this also depends on the access to pasture [86].

Stress is considered another factor that influences MSCC, leading to higher values. Various practices such as blood sampling, tuberculosis skin tests, vaccinations or transport lead to a temporary decrease in milk production and an increase in MSCC [79,87].

Furthermore, another parameter that should be taken into account when evaluating and interpreting MSCC in goat’s milk is the alternation of seasons or seasonality. Thus, warm season is associated with an increase in milk production and consequently lower cell count value, while in the cold months, the MSCC increases along with a reduction in milk production [88,89].

Multiple attempts have been made to establish threshold values for MSCC in goat’s milk; in the past a limit of 750 × 10^3^ cells/mL was suggested as a predictive value for infection with minor pathogens and a value of 1750 × 10^3^ cells/mL for major pathogens [90], whereas other authors proposed a limit of 345 × 10^3^ cells/mL to differentiate a healthy udder from an infected one [76]. Most studies aimed at identifying intramammary infections in goats have suggested a range of 500 to 1000 × 10^3^ cells/mL milk for an infected mammary gland [73,79,90,91].

Among the bacterial pathogens involved in the etiology of mastitis in goats, *Staphylococcus aureus* is proven to be responsible for the most significant increase in MSCC, up to 3,000,000 cells/mL, leading to much higher values compared to NAS infections [3,12,79,92,93]. However, mastitis caused by NAS are also characterized by marked increases in MSCC, especially in the case of *Staphylococcus epidermidis*, *Staphylococcus caprae*, *Staphylococcus simulans* and *Staphylococcus xylosus* [23]. In cows, NAS mastitis is known to moderately increase MSCC, but not in such a great extent as in small ruminants, goats or sheep [3,13]. Moreover, other bacterial agents responsible for significantly increased values of this parameter are *Trueperella pyogenes* (formerly known as *Arcanobacterium pyogenes* or *Corynebacterium pyogenes*), as well as *Mycoplasma agalactiae* [42]. Regarding coliform mastitis, the MSCC has a low value before and after the installation of clinical signs, while in the case of *S. aureus* infection, MSCC increases before the clinical phase and remains at a high level even after healing. The same observation is available for mastitis caused by streptococci [6,94]. Therefore, given the above presented aspects and the physiological features of the mammary gland in goats, MSCC must be correlated with other indicators in order to establish the diagnosis of an intramammary infection.

## 3. Oxidative Stress

### 3.1. Reactive Oxygen Species (ROS), Reactive Nitrogen Species (RNS) and Antioxidant Systems

Oxidative stress is defined as an imbalance between the production of reactive oxygen species (ROS) and the antioxidant mechanisms of the organism [95]. Due to the existence of a very wide range of enzymes and compounds with both pro- and anti-oxidant activity, oxidative stress was classified on a scale ranging from physiological oxidative processes (eustress) to toxic oxidative phenomena (distress) [96]. Free radicals are defined as unstable molecules that contain one or more unpaired electrons, which increase their reactivity. Examples of such free radicals are the hydroxyl radical, the superoxide anion, nitric oxide and peroxynitrite [95,97]. Free radicals occur as a result of various biochemical processes that take place in the body. Some examples in this regard would be the reduction of molecular oxygen during aerobic respiration and the oxidation of catecholamines, processes that lead to the formation of superoxide and hydroxide radicals [95]. Moreover, macrophage activation is another process that causes superoxide to form, whereas vascular endothelium is responsible for producing NO. These free radicals can also be synthesized in response to various external factors, such as electromagnetic radiation, which break down water to form hydroxyl radicals. If the antioxidant mechanisms are overwhelmed, the organism’s homeostasis is affected, with serious consequences in various tissues and organs [95,98].

The superoxide anion is an unstable species, which is formed under normal conditions in the body following the process of incomplete reduction of the oxygen molecule, especially in the mitochondrial respiration chain, but also following the activity of cells with phagocytic capacity [98,99]. Such cells are represented by macrophages, neutrophils or monocytes. During a bacterial infection, a large amount of superoxide anion is produced, being involved in the process of destruction and elimination of pathogenic microorganisms. Cytochrome P450 is another significant source of superoxide anion. This cytochrome belongs to the category of monooxygenases and is present in the endoplasmic reticulum [99]. It is estimated that approximately 10^10^ superoxide molecules form in the animal body within 24 h [100]. Not all superoxide anions initiate autooxidation and peroxidation of molecules; only a small amount of them do this. Most anions are constantly converted to hydrogen peroxide under the action of an enzyme called superoxide dismutase (SOD). This reaction involves the transfer of an electron to another superoxide molecule and the incorporation of a proton [98]. Hydrogen peroxide is broken down by specific enzymes of the hydroperoxidase class, such as catalase and peroxidases, mainly glutathione peroxidase. The activity of these enzymes prevents the excessive accumulation of hydrogen peroxide in the tissues, a harmful phenomenon to the organism. However, it is considered that the presence of hydrogen peroxide in low amounts is of great importance due to involvement in certain physiological processes, such as unsaturated fatty acid metabolism, phagocytosis or the immune response [99].

The hydroxyl radical is another ROS, and it is actually the most reactive radical in the body, with a very short half-life [96,98]. It is formed following the reaction of superoxide with hydrogen peroxide in the presence of transition metals ions (iron, copper). Hydroxyl radical can damage proteins, lipids, carbohydrates. It can also start lipid peroxidation by taking an electron from polyunsaturated fatty acids, and it is also involved in the oxidative degradation of nucleic acids [95,99].

Other oxygen-derived free radicals are the peroxyl radicals, and the simplest form of these radicals is hydroperoxyl radical, playing a role in fatty acid peroxidation [98]. The peroxyl radical is formed following the action of other ROS on lipoproteins and fatty acids [99].

The targets of oxidative reactions in the presence of ROS are mainly nucleic acids, proteins and lipids. Oxidation of proteins leads to changes in the enzymatic activity, with consequences on metabolic processes. On the other hand, lipids are an important component of the cell membrane, and these oxidative processes mainly affect unsaturated fatty acids (due to the presence of double bonds) that can initiate chain reactions which compromise cell integrity [97].

In addition to ROS, special attention has recently been paid to reactive nitrogen species (RNS), which include nitric oxide (NO) and its metabolites, which are involved in various physiological and pathological phenomena that occur in the animal body [99]. Nitric oxide (NO) is a gaseous, inorganic, uncharged diatomic molecule which contains an unpaired electron that determines its reactivity to other molecules, such as glutathione, oxygen or the superoxide radical [101]. This molecule is synthesized at cellular level due to the action of specific enzymes called nitric oxide synthetases, L-arginine being their substrate [99]. Over time, NO has been considered one of the most versatile “players” of the immune system. It is involved in the pathogenesis and control of infectious diseases, tumors, autoimmune and degenerative diseases. NO’s role in the immune system is not fully understood, because both protective effects and toxic effects are frequently observed [102]. Nevertheless, any claimed biological action of NO should be connected with its interaction with kinetically relevant biotargets [101]. Although NO is not considered toxic, many of its effects at the cellular level are mediated by its oxidation products. NO production is a characteristic of immune cells (dendritic cells, natural killer cells, mast cells, monocytes, macrophages, Kupffer cells, neutrophils, and eosinophils), but these molecules can also be synthesized by other cells, such as endothelial cells, epithelial cells, keratinocytes, chondrocytes or hepatocytes. Thus, NO has antimicrobial, antitumor, pro- or anti-inflammatory activity [102]. The antibacterial, cytotoxic and inflammatory effects of NO are independent of cGMP and may occur as a result of the interaction of this molecule with thiol groups, transition metals or free radicals, such as the superoxide anion. NO has a very short half-life, hence the high affinity for it of the superoxide anion, which reduces the bioavailability of NO [99].

Peroxynitrite is formed as a result of the reaction between NO and the superoxide anion. Normally, this molecule is unstable, being easily isomerized to nitrate. Nitrate is biochemically inert in animal cells, so NO is considered to be an antioxidant because it inactivates the superoxide anion to some extent. However, the accumulation of large amounts of peroxynitrite is harmful to the organism due to the oxidative effect on tissues, which leads to oxidation of proteins and peroxidation of lipids [99].

Superior organisms have antioxidant systems which prevent the excessive accumulation of ROS. These systems are divided into two broad categories, enzymatic antioxidants on the one hand, and nonenzymatic antioxidants on the other [98]. The enzymatic antioxidant system is located at the cellular level in different compartments. Antioxidant enzymes are represented by superoxide dismutase (SOD), peroxidases, catalase, hemoxygenase etc. On the other hand, nonenzymatic antioxidant systems are represented by molecules that have the ability to oxidize in the presence of ROS, thus protecting structural compounds against oxidation. These nonenzymatic antioxidants are classified according to their solubility in fat-soluble antioxidants and water-soluble antioxidants. The first category includes vitamins A and E, carotenoid pigments and ubiquinones (coenzyme Q), whereas water-soluble antioxidants include vitamin C, cysteine, glutathione, uric acid, bilirubin, etc. Other molecules with antioxidant roles are some amino acids (histidine, taurine), but also metals such as selenium [98,99].

### 3.2. Changes in Oxidative Stress Indices in Goat Mastitic Milk

Many infectious diseases of farm animals are thought to be associated with oxidative stress. Thus, in the pathophysiology of certain diseases, oxidative stress seems to play an important role. Sepsis, pneumonia or enteritis in various animal species (bovine, pigs), as well as recurrent obstructive respiratory disease in horses are conditions in which the involvement of ROS is proven, affecting the balance between pro- and antioxidant activity. Moreover, in the case of mastitis, changes caused by oxidative stress can be observed in the udder tissue, but also in the blood. These aspects have been studied over time, with numerous studies focused especially on cow’s milk [8,97]. During inflammatory processes in the mammary gland, an accentuated lipid peroxidation can be observed, which leads to a decrease in molecules with antioxidant role, with the consequent installation of oxidative stress [103].

Measuring oxidative stress can be achieved by various methods, such as direct determination of ROS and RNS; assessment of total antioxidant capacity and activity of enzymatic antioxidant systems; determination of byproducts resulting from oxidation of proteins, lipids and nucleic acids; and determination of the oxidation degree of endogenous substances such as vitamin E, glutathione, carotenoids and ascorbic acid [99].

Milk has its own antioxidant systems that play a particularly important role in preventing lipid peroxidation and maintaining its quality. Thus, the antioxidant activity of milk is due to the presence of certain enzymes, such as catalase, lactoperoxidase, glutathione-peroxidase, xanthine oxidase or nonenzymatic compounds, especially vitamins and provitamins (retinoids, carotenoids, tocopherol, ascorbic acid) [104]. Intramammary infections have consequences on the enzymatic and nonenzymatic antioxidant activity, therefore, the activity of some compounds can be used as indicators of mastitis [7,8]. In general, intramammary infections are associated with a reduced milk antioxidant capacity [27,103].

Catalase is an oxidoreductase, which catalyzes the degradation of hydrogen peroxide with the formation of water and oxygen. This molecule is also present in milk, and its activity depends on several factors such as diet, lactation or the presence of intramammary infections; thus, it can be considered an indicator of mastitis [34]. Catalase plays a key role in the milk redox system, which correlates with the increase in enzymatic activity in the case of intramammary infections in cows [8]. In raw cow milk, the average catalase activity is 1.96 U/mL, a value obtained using a polarimetric method, but there are variations of this value depending on the diet, the stage of lactation or the evolution of an inflammatory process. In a study focused on the association of subclinical mastitis and oxidative stress in goats, it has been demonstrated that subclinical intramammary infections lead to a significant decrease in the antioxidant capacity of milk, especially those caused by NAS. The same authors showed that in healthy goat’s milk, the value of catalase activity is 3.8 U/mL, higher than the value established for cattle, 2.1 U/mL [27]. Catalase, in addition to the decomposition of hydrogen peroxide, also intervenes in the NO cycle by transforming nitrite into nitrate, a less active form. Oxidation of nitrite to nitrate is necessary to prevent the accumulation of free radicals and oxidation products during milk stasis in the mammary cistern, as well as during the period between milking and pasteurization [105]. Thus, catalase is one of the key enzymes in the prevention of excessive nitrosative stress in milk [8,27,105].

Lactoperoxidase (LPx) is one of the most abundant enzymes in milk and is considered to be part of the mammary gland immune system, with both bactericidal and bacteriostatic effects in milk. This enzyme plays a major role in preventing intramammary infections, along with xanthine oxidase (XO) and NO [47,106]. In mastitic bovine milk, the activity of lactoperoxidase is increased, this increase being correlated with a high MSCC [34]. Along with the activity of LPx, the activity of another milk enzyme called glutathione peroxidase (GPx) increases, but these two molecules intervene at different moments of the inflammatory process. Thus, lactoperoxidase is responsible for increasing oxidative stress and antibacterial resistance in milk, whereas GPx intervenes in the process of oxidative stress resolution [8]. Similar to cow milk, the LPx activity in goat milk increases with the increase in MSCC, as this enzyme is mainly synthesized by PMN [107]. Therefore, in addition to its antimicrobial properties, LPx can also be used to detect subclinical mastitis in dairy goats. In a study performed on two different breeds of goats (Saanen and South African indigenous goats), it has been demonstrated that a subclinical inflammatory process leads to a significant increase in LPx, a phenomenon which is directly proportional to MSCC [107]. It is worth mentioning that goat breeds that physiologically secrete a large number of somatic cells in milk will have a higher LPx activity, even in the absence of an intramammary infection. Therefore, the interpretation of the results should be conducted in a clinical context, together with other indicators (enzymes). Furthermore, breed is a factor which also influences the antibacterial activity of LPx, as experimental studies show [108]. For example, LPx from Saanen goat milk had a strong bactericidal effect on a strain of *Staphylococcus aureus*, whereas the same enzyme from South African indigenous goats had only a bacteriostatic effect on the same bacterial strain. These results support the hypothesis that LPx is involved in local antibacterial defense mechanisms [108]. Moreover, this enzyme is characterized by thermal stability, so it can be used as an index of milk pasteurization efficiency [34].

Glutathione peroxidase (GPx) is another important antioxidant enzyme, whose activity changes during intramammary infections. GPx is widespread in the cytoplasm of animal cells, and its role is to protect cells against the damaging effects of peroxides, breaking down peroxides in the presence of glutathione. The active site of this enzyme contains selenium in the form of selenium-cysteine. There are two different classes of GPx, selenium-dependent and selenium-independent GPx. The first category includes enzymes that have the ability to break down hydrogen peroxide [7]. In cows with subclinical mastitis, the activity of this enzyme in milk has been shown to be much higher compared to milk from healthy animals. Moreover, the level of enzymatic activity depends on the pathogen involved in the etiology of mastitis [104]. Thus, experimentally inoculated cow milk with a strain of *Escherichia coli* showed a 10 times higher GPx activity compared to healthy milk. Moreover, the obtained values were higher compared to *Streptococcus viridans*, *Staphylococcus aureus*, *Pseudomonas aeruginosa* or *Candida* spp. infection. GPx activity also correlates with MSCC, being directly proportional to it [7]. The same observations were made for goat’s milk. In addition to GPx, along with the increase in MSCC, the concentrations of other enzymes also increase, such as lipase, phosphatase, lactate dehydrogenase, xanthine oxidase or catalase [107].

Xanthine oxidoreductase is an enzyme which catalyzes the oxidation reaction of xanthine to hypoxanthine, with the simultaneous reduction of oxygen and the formation of hydrogen peroxide. The two forms of xanthine oxidoreductase are xanthine oxidase (XO) and xanthine dehydrogenase (XDH). Following proteolysis reactions, XDH turns into XO. Structurally, XO is a metalloflavoprotein, being a major component of milk fat globules [34,106]. In milk, this enzyme plays different roles. Thus, XO has a microbicidal activity, but it is not very accentuated due to its ability to form superoxide and hydrogen peroxide, the latter being used as a substrate by LPx. Studies have shown that the bactericidal effect in milk is also due to the formation of NO. XO converts nitrate to nitrite, this process significantly increasing the substrate required for NO generation. Therefore, XO is considered to be part of the mammary gland’s immune system, along with LPx and NO [47].

The presence of superoxide dismutase (SOD) in milk is essential in order to maintain its antioxidant stability. In cow’s milk, this enzyme has the same activity, molecular weight and the same electrophoretic properties as Cu, Zn-SOD found in bovine erythrocytes [34]. An increased SOD concentration is associated with the presence of free radicals and oxidative processes in milk. It seems that in goats, the postpartum period influences the milk activity of SOD. Thus, one study showed that in the first three weeks after calving, the concentration of this enzyme was rather low, this indicating a weak ROS activity in this period, simultaneously with an increase in total antioxidant status. Starting with the fourth week, the activity of SOD and oxygen radicals increases, but there are not enough data to explain this phenomenon [109].

Ascorbic acid is a water-soluble nonenzymatic antioxidant, which counteracts the harmful effects of ROS in the organism. Ascorbic acid is synthesized by plants and animals, except for humans, primates, guinea pigs, several birds and fish, being an indispensable molecule in the body with an important antioxidant role [8]. Goat’s milk contains on average 1.29 mg/100 g of ascorbic acid, more than cow’s milk (0.94 mg/100 g) [30]. The amount of ascorbic acid decreases during pathological processes, for example in mastitis, but this decrease can also occur after heat treatment and ultraviolet or oxygen exposure during milk processing [8,110]. Moreover, in milk containing a large number of somatic cells, the concentration of antioxidant vitamins is lower compared to milk with a low MSCC [111]. Thus, in the case of subclinical mastitis, there is a pronounced reduction in the concentration of vitamin C; this is due to the fact that ascorbic acid is consumed during oxidative processes. In a healthy goat mammary gland, the concentration of vitamin C in the milk is higher by 36.4% compared to an infected udder tissue [27].

Vitamin E represents a group of compounds, tocopherols and tocotrienols, which are exclusively synthesized by plants, the most common being α-tocopherol. These molecules are characterized by a very effective antioxidant activity, especially in cell membranes [34,111]. Goat’s milk contains on average 0.04 mg/100 g of vitamin E, its concentration depending on diet, season or stage of lactation [30]. Certain procedures such as milk skimming or heat treatments lead to a significant decrease in the concentration of these vitamins, as well as ultraviolet exposure [110].

Vitamin A belongs to the category of fat-soluble vitamins and it is found in animal organisms in three forms: retinol, retinal and retinoic acid. Goat milk is rich in vitamin A, with about 185 IU/100 g, more than cow’s milk [30]. An important role is played by carotenoids, which function as antioxidants and immunostimulants and prevent degenerative and cardiovascular diseases. The stability of retinol and carotenoids in milk depends on how they are processed. It should be noted that these compounds are sensitive to light and temperature, as studies performed on goat and cow milk show [110]. Thus, UV exposure leads to a decrease in the level of retinol in milk inversely proportional to the concentration of fat, which suggest that lipids do have a protective role [34]. Moreover, intramammary infections lead to a decrease in the concentration of retinol and carotenoids in cow’s milk, with effects on their antioxidant capacity [8]. High MSCC also affects the retinol concentration. Thus, a MSCC higher than 500,000/mL causes a decrease in retinol levels, along with a decrease in α-tocopherol concentration [111]. Data regarding changes in vitamin A concentrations in mastitic goat milk are still scarce.

As previously presented, NO is part of the mammary gland’s immune system, having antimicrobial activity (bactericidal and bacteriostatic) and a protective effect against infections that could occur during milk stasis in the mammary gland [47]. Milk is considered a very complex system from a biochemical and biophysical point of view, because hydrogen peroxide and NO are constantly produced in mammary epithelial cells and somatic cells due to the activity of certain enzymes, such as XO, SOD and nitric synthetases [105]. NO has cyclic activity in milk due to the succession and repetition of certain reactions. This cyclicity was initially demonstrated in cow’s milk, then also confirmed for goat’s milk. Hydrogen peroxide plays an essential role in this cycle, because this compound is used as a substrate for lactoperoxidase, but also for catalase in the reaction of nitrite to nitrate [27]. Clinical and subclinical mastitis are associated not only with oxidative stress, but also with nitrosative stress, caused by the formation and accumulation of RNS. Bacterial infectious processes are associated with an increase in the concentration of NO, nitrite, nitrate and other oxidation compounds produced by mammary cells [8]. Intramammary inoculation of an *E. coli* strain or lipopolysaccharide in cattle leads to a marked increase in nitrite and nitrate levels in the blood and milk of cows studied. Moreover, in cows with subclinical mastitis caused by *Staphylococcus aureus*, *Streptococcus agalactiae* or *Escherichia coli*, a significant decrease in NO in both milk and serum is observed compared to healthy animals. The same observations were made for acute forms of mastitis [97]. Research that aimed to establish a correlation between subclinical mastitis in goats caused by NAS and the onset of nitrosative stress showed that milk from an infected mammary gland is characterized by high levels of nitrites and nitrates, along with a decrease in milk production and lactose concentration. Thus, a moderate nitrosative stress was present and along with it a significant reduction of the total antioxidant status and the level of milk ascorbic acid [27].

Another interesting phenomenon with great importance in the pathogenesis of intramammary infections is the ability of pathogens to survive and multiply under oxidative stress conditions. During phagocytosis, macrophages and other immunologically competent cells generate superoxide and other ROS involved in antibacterial activity [7]. Many microorganisms have their own antioxidant systems, represented by SOD and catalase, which explains their ability to survive. Moreover, studies have shown that in infections caused by *Streptococcus agalactiae*, this microorganism synthesizes its own glutathione, a molecule that plays an important role in protecting the bacterial cell against oxidative stress [112]. The resistance of bacteria to hydrogen peroxide is dependent on the accumulation of glutathione, which activates the glutathione-peroxidase–glutathione-reductase system in the cells [7].

Due to the difficulty in evaluating the individual activity of antioxidant compounds, different protocols have been developed in order to measure total antioxidant capacity (TAC) or total antioxidant status (TAS). There are two categories of methods for determining TAC, methods based on hydrogen transfer and techniques based on electron transfer. Thus, the evaluation of this parameter includes the cumulative activity of all antioxidants found in plasma or other secretions, and can be considered an integrative parameter [99,103]. TAC in goat milk is proven to be higher compared to the milk of other species, such as cattle, donkeys, humans, sheep or bison. This is also due to the higher amount of ascorbic acid in its composition. Due to these particularities, research has shown that a constant consumption of milk from goat breeds with a high level of TAC, such as the Greek breed Prisca, significantly increases the level of this parameter in the blood of the consumer [27]. Moreover, studies on Aardi goats showed that the TAC in milk varies with the stage of lactation, having higher values in the second and third week postpartum, and decreasing significantly in the fourth week after calving [109]. Similar results were obtained for an Italian breed in the Umbria region [113]. Breed, diet and season are factors that influence the milk TAC, but currently there are not enough data to explain the mechanisms by which these factors alter the antioxidant activity of goat milk [114]. In cows with subclinical mastitis, the milk TAC is significantly lower compared to milk from healthy cows, which leads to the accumulation of ROS and the installation of oxidative stress. Thus, milk from an affected mammary gland contains a higher concentration of compounds resulting from oxidative degradation of lipids and proteins [8,27,115]. The same observations were made for the goat species [27].

The effects of oxidative stress can be observed at multiple levels, ROS producing irreversible oxidation changes of biomolecules. Lipids represent a category of molecules which undergo peroxidation processes, with the formation of hydroperoxides and peroxide radicals of lipids [98]. At the cellular level, changes in the bilipid membrane may occur, with inactivation of membrane receptors and increased tissue permeability. The most susceptible lipids to self-oxidation are those with a high content of unsaturated fatty acids [98,99]. Proteins can also suffer from oxidative degradation at the level of amino acid residues in polypeptide chains, processes followed by enzymatic inactivation and increased susceptibility to various phenomena of precipitation or proteolysis. It has been shown that an increase in the degree of oxidation of polyunsaturated fatty acids leads to an increase in protein oxidation. This has been highlighted by the increase in the concentration of carbonyl compounds resulting from the oxidation processes of amino acids [99]. ROS can cause changes in nucleic acids, which result in mutations, depletions or translocations. Most of these changes are of major relevance in the process of carcinogenesis, aging, neurodegenerative diseases, cardiovascular and autoimmune diseases [98]. Following these oxidative phenomena, hydroperoxides and peroxide radicals of the nucleic bases are formed, which lead to the modification of DNA strands and its defective replication [99]. Moreover, regarding milk fat, the phenomena of lipid peroxidation lead to the formation of products, lipid hydroperoxides that decompose and form other compounds called reactive carbonyl species. The most important are aldehydes, dialdehydes (malonyldialdehyde and glyoxal), ketoaldehydes, furans and short-chain hydrocarbons. The most common method to determine the degree of lipid peroxidation is malonyldialdehyde (MDA) dosing. This can be measured using spectrophotometric methods, based on the reaction with thiobarbituric acid, or chromatographic methods. Additionally, other products such as isoprostanes can be used as indirect markers of oxidative stress, along with the quantitative determination of MDA [98]. In mastitic milk, the concentration of MDA is higher than in healthy milk, demonstrating that during infectious processes there is an accumulation of ROS and products resulting from oxidative degradation of biomolecules [8]. Furthermore, increased MSCC may be correlated with an increase in MDA levels and a decrease in the proportion of unsaturated fatty acids, as studies conducted on bovine milk show [34]. Research focused on goat milk lipid peroxidation is yet to be published.

## 4. Conclusions

Mastitis is responsible for producing certain changes regarding both milk quantity and quality. Such changes are visible in large and small ruminant milk, including goat, but it is worth mentioning that in goat species, there are more numerous factors which influence milk composition compared to cows, and these aspects should be taken into account whenever interpreting test results or establishing a mastitis diagnosis. In general, the inflammation of the mammary gland has negative consequences on milk production, as well as on milk biochemical parameters. Therefore, studies have reported a significant increase in whey proteins and albumin concentration during mastitis, but major changes are observed in casein proportion due to pronounced hydrolysis following plasmin activity. Moreover, goat mastitic milk is characterized by a reduced lactose concentration and milk fat, although results concerning the latter are contradictory. The concentration of certain enzymes also changes once the pathological process is installed. Therefore, the activity of indigenous enzymes increases during inflammation, whereas enzymes involved in the process of milk synthesis have a lower activity. Intramammary infections are also responsible for modifying the milk electrical conductivity, a parameter currently used in cattle in the detection of subclinical mastitis. In goats, the electrical conductivity of the mastitic milk is increased, especially when both halves of the mammary gland are affected. Furthermore, MSCC in goats is a valuable indicator of the inflammatory process in the mammary tissue, but this parameter is strongly influenced by numerous intrinsic and extrinsic factors. This is the reason why MSCC results should always be evaluated in a clinical context. Nevertheless, mastitis is also associated with the oxidative and nitrosative stress phenomena due to the accumulation of ROS and RNS, which cause cell and tissue damage, affecting the milk antioxidant properties. In conclusion, milk biochemical parameters, oxidative and nitrosative stress indices in goats are significantly influenced by infectious processes, but more research is needed in order to establish strong correlations between changes in milk components and pathogenic agents, as well as new diagnostic methods for goat mastitis relying on relevant inflammation markers.
